# No significant difference in intermediate key outcomes in men with low- and intermediate-risk prostate cancer managed by active surveillance

**DOI:** 10.1038/s41598-022-10741-8

**Published:** 2022-04-25

**Authors:** Karolina Cyll, Sven Löffeler, Birgitte Carlsen, Karin Skogstad, May Lisbeth Plathan, Martin Landquist, Erik Skaaheim Haug

**Affiliations:** 1 Department of Urology, Vestfold Hospital Trust, NO-3103 Tønsberg, Norway; 2 Institute for Cancer Genetics and Informatics, Oslo University Hospital, NO-0424 Oslo, Norway; 3 Department of Pathology, Vestfold Hospital Trust, NO-3103 Tønsberg, Norway; 4 Department of Radiology, Vestfold Hospital Trust, NO-3103 Tønsberg, Norway

**Keywords:** Medical research, Oncology, Urology

## Abstract

Active surveillance (AS) is standard of care for patients with low-risk prostate cancer (PCa), but its feasibility in intermediate-risk patients is controversial. We compared outcomes of low- and intermediate-risk patients managed with multiparametric magnetic resonance imaging (mpMRI)-supported AS in a community hospital. Of the 433 patients enrolled in AS between 2009 and 2016, 358 complied with AS inclusion criteria (Cancer of the Prostate Risk Assessment (CAPRA) score ≤ 5, Gleason grade group (GGG) ≤ 2, clinical stage ≤ cT2 and prostate-specific antigen (PSA) ≤ 20 ng/ml) and discontinuation criteria (histological-, PSA-, clinical- or radiological disease reclassification). Of the 358 patients, 177 (49%) were low-risk and 181 (51%) were intermediate-risk. Median follow-up was 4.2 years. The estimated 5-year treatment-free survival (TFS) was 56% (95% confidence interval [CI] 51–62%). Intermediate-risk patients had significantly shorter TFS compared with low-risk patients (hazard ratio 2.01, 95% CI 1.47–2.76, p < 0.001). There were no statistically significant differences in the rate of adverse pathology, biochemical recurrence-free survival and overall survival between low- and intermediate-risk patients. Two patients developed metastatic disease and three died of PCa. These results suggest that selected patients with intermediate-risk PCa may be safely managed by mpMRI-supported AS, but longer follow-up is necessary.

## Introduction

Prostate cancer (PCa) is the second most commonly diagnosed cancer and fifth leading cause of cancer-related death in men worldwide^1^. Approximately 80% of men are diagnosed with localized disease, which is classified into low-risk (LR), intermediate-risk (IR) and high-risk groups, based on prostate specific antigen (PSA) levels, tumor stage and histological assessment of Gleason grade group (GGG)^2^.

The standard curatively-intended treatments for localized PCa—radical prostatectomy (RP) and radiation therapy—often result in adverse side-effects that can reduce quality of life, including urinary incontinence, bowel and erectile dysfunction^3^. Active surveillance (AS) is an alternative option to immediate treatment, developed in response to the concerns about overtreatment of PCa. AS aims to avoid or at least delay the start of treatment, without compromising survival and quality of life. Men enrolled in AS are regularly monitored with prostate biopsies, PSA measurements, rectal exams and multiparametric magnetic resonance imaging (mpMRI). Treatment is recommended when the disease is reclassified as higher risk, defined as e.g. increase in GGG or tumor stage^4^.

Currently, AS is a standard of care for patients with LR PCa^5,6^. It is disputed whether patients with IR disease can be safely managed with AS^6^. Although IR patients have more mixed oncological outcomes in observational studies compared with LR patients^7–9^, the majority would likely benefit from AS rather than undergoing immediate treatment^10^. Importantly, contemporary IR patients present with less aggressive tumors than those in historical cohorts due to modifications of the Gleason grading system^11^. Additionally, use of mpMRI and mpMRI-targeted biopsies regularly results in upgrading and upstaging of tumors^12,13^. The net result is an increase in the number of patients diagnosed with IR disease, fewer occult aggressive tumors within the IR group and likely a better prognosis^13,14^.

Published AS cohorts have reported 13–30%^15–22^ patients with IR disease based on various modifications of D’Amico classification or the Cancer of the Prostate Risk Assessment (CAPRA) score. Dall’Era and Klotz reviewed the results from five such cohorts with median follow-up up to 6.4 years and concluded that although IR patients were more likely to receive treatment, their chance for long-term cure was not significantly compromised^23^. However, most of these studies were performed at tertiary centers^16,18,19^, inclusion and follow-up of IR patients was often not pre-planned^15–17,22^ and mpMRI was not regularly used^15–21^. Also, in some of the studies the outcomes were not stratified between LR and IR groups^18,20^.

In this study, we describe outcomes from a non-academic, population-based AS cohort, which includes both LR and IR patients. Follow-up involved mpMRI scans and mpMRI-targeted biopsies in addition to standard measurements. The aim of this study was to compare treatment-free survival (TFS) and post-treatment outcomes in LR and IR patients. In addition, we studied changes in Gleason grade group (GGG), PSA levels and Prostate Imaging–Reporting and Data System (PI-RADS) score during AS in both patient groups.

## Patients and methods

The study was approved by the Regional Committee for Medical and Health Research Ethics (REC) in Norway (REC no. 2012/1679). A broad informed consent was obtained from all study participants.

Between August 2009 and December 2016, 433 consecutive patients were diagnosed with PCa and enrolled in the AS program at Vestfold Hospital Trust (VHT). VHT provides PCa care for all patients in Vestfold County (Population 240,000, the average annual number of new PCa cases 2013–2017: 263) including diagnosis, treatment and follow-up, allowing for access to almost complete patient data. During this time period, 27 (13%) of 209 patients diagnosed with LR and 139 (43%) of 322 patients diagnosed with IR disease received immediate treatment (Table S1). AS protocol used at VHT is detailed in Table 1. The upper age limit for offering active surveillance was changed from 75 to 80 years in 2014. AS inclusion and discontinuation in all patients were based on recommendations by a multidisciplinary team and shared decision-making.

**Table 1:** Active surveillance protocol at Vestfold Hospital Trust.

AS inclusion criteria	Follow-up scheme	AS discontinuation criteria
Age < 75 years^a^	**Low-risk:**	**Triggers for treatment:**
GGG < 3	PSA every 3 months during the first year and every 6 months thereafter	Histological reclassification (GGG ≥ 3, perineural invasion or increase in number of positive biopsies)
PSA ≤ 20 ng/ml	Repeat biopsy^c^ 12 after AS enrollment and every 60 months thereafter (or at increase of PSA level or tumor size)	PSA reclassification (PSA > 20 ng/ml^§^ or PSA doubling time < 1 year)
cT < 3	MRI^†^ after 12, 48 and 60 months	Clinical reclassification (cT ≥ 3)
Life expectancy > 5 years^b^	**Intermediate-risk:**	Radiological reclassification (EPE or SVI, increase in tumor diameter or the number of PI-RADS score > 3 lesions)
Patient preference	PSA every 3 months during the first 2 years and every 6 months thereafter	Patient preference
**Low-risk:**	Repeat biopsy^c^ 12 and 24 after AS enrollment, and every 60 months thereafter (or at increase of PSA level or tumor size)	**Transferal to watchful waiting:**
CAPRA 0–2 and PSA < 10 ng/ml and GGG 1	MRI after 12, 24 and 48, and every second year after that	Age ≥ 75^a^
**Intermediate-risk:**		Life expectancy ≤ 5 years^b^
CAPRA 3–5 and/or PSA 10–20 ng/ml and/or GGG 2		

*AS* active surveillance, *CAPRA* cancer of the prostate risk assessment, *EPE* extraprostatic extension, *GGG* Gleason grade group, *MRI* magnetic resonance imaging, *PSA* prostate-specific antigen, *SVI* seminal vesicle invasion.

^a^Changed from 75 to 80 years in 2014.

^b^Based on Charlson comorbidity index.

^c^Systematic and/or MRI-targeted.

^d^On at least two consecutive measurements.

**Figure 1:**
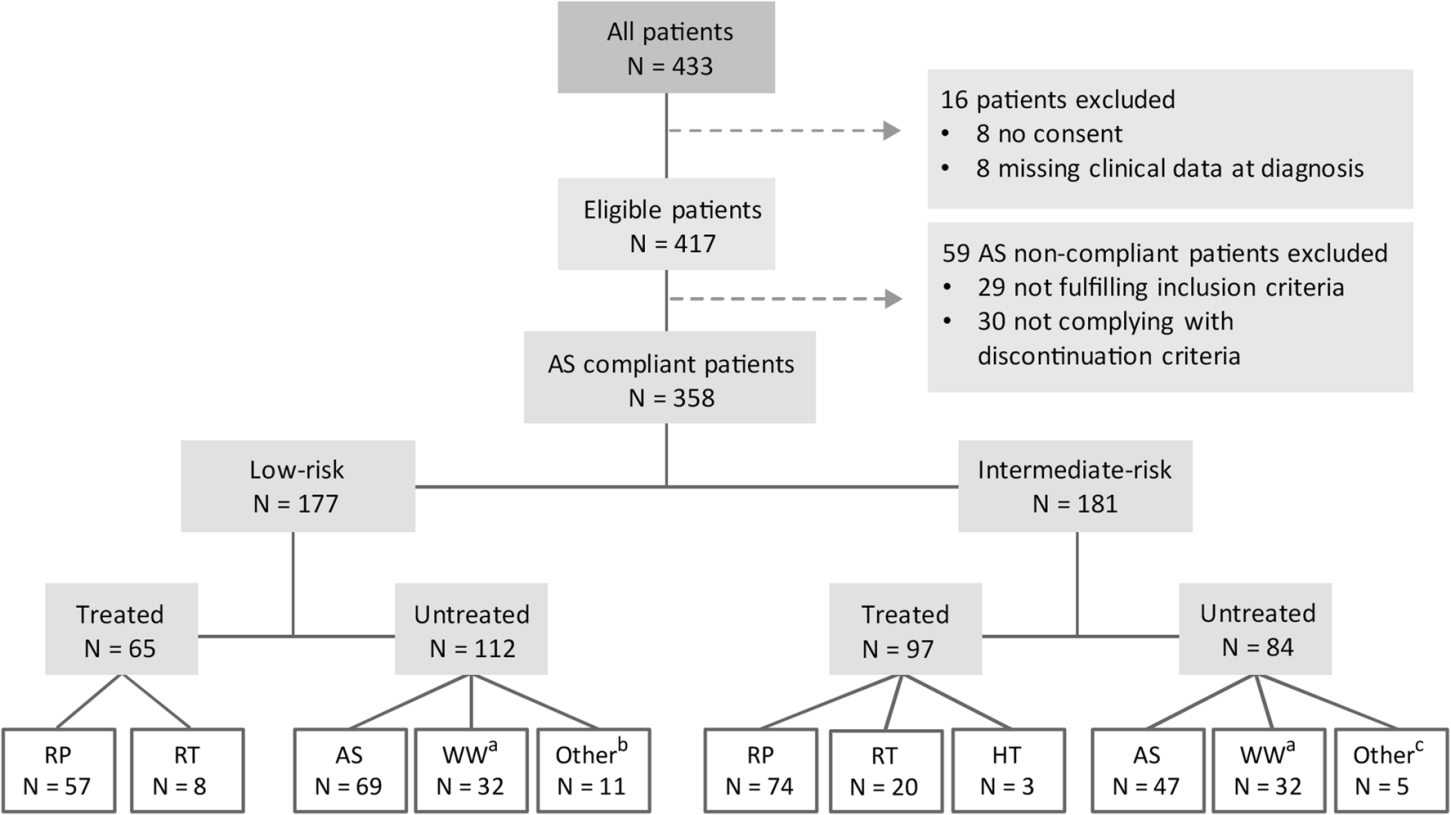
CONSORT diagram for the Vestfold Hospital Trust active surveillance program. *AS* active surveillance, *HT* hormonal therapy, *RP* radical prostatectomy, *RT* radiotherapy, *WW* watchful waiting. ^a^Four intermediate-risk patients and two low-risk patients had disease reclassification but were transferred to WW due to low life expectancy; ^b^Nine patients were lost to follow-up and two died; ^c^ Four patients were lost to follow-up and one died.

At diagnosis, patients were classified as LR (CAPRA ≤ 2 and prostate specific antigen [PSA] < 10 ng/ml and GGG 1) or IR (CAPRA 3–5 or PSA 10–20 ng/ml or GGG 2). Patients diagnosed with IR disease had more regular follow-ups than those diagnosed with LR disease (Table 1). Patients reclassified from LR to IR during follow-up received more regular biopsies and/or mpMRI scans at the urologist discretion. Patients who did not provide a broad informed consent at diagnosis (n = 8) and those missing baseline data (n = 8) were excluded, while those managed by AS despite not fulfilling the inclusion criteria or not complying with the AS discontinuation criteria were designated non-compliant (n = 59; Fig. 1).

Primary endpoints of this study were TFS and adverse pathology (AP) after RP. Two definitions of AP were used; a less severe AP1 (GGG ≥ 3 or pT stage ≥ pT3a or pN1) and a more severe AP2 (GGG ≥ 4 or pT stage ≥ pT3b or pN1). Secondary endpoints were biochemical recurrence (BCR), defined as PSA ≥ 0.2 ng/ml at least 6 weeks after RP or a rise by 2 ng/mL or more above the nadir PSA after radiotherapy (RT), and overall survival (OS). Time to BCR was calculated from initiation of curative treatment (surgery or RT) to BCR or to the date of the final PSA registration. Time to event in the analyses of TSF and OS was calculated from the date of PCa diagnosis to event or 31st of December 2020 if still in AS at that time. Life tables and Kaplan–Meier survival curves were applied for TFS, BCR and OS outcomes. Survival distributions were compared using the Mantel–Cox log-rank test in univariable analysis of categorical variables and the Wald’s chi-squared test in univariable analysis of continuous variables and in multivariable analyses. Hazard ratios (HR) and 95% confidence intervals (CI)s were calculated using the Cox proportional hazard model. Multivariable analysis of BCR included risk group at diagnosis and well-established clinicopathologic characteristics at the time of primary surgery, whereas multivariable analysis of TFS and OS included risk group and clinicopathologic characteristics at diagnosis, except for those used to define risk groups. Descriptive statistics were used for patient characteristics; median and interquartile ranges (IQRs) were used for continuous variables, and frequencies and percentages for categorical variables. Associations were evaluated using the Fischer’s exact test for categorical variables and Mann–Whitney’s *U*-test for continuous variables. Two-sided p-values < 0.05 were considered statistically significant. Statistical calculations were performed using Stata/SE 16.1 (StataCorp, College Station, TX, USA).

### Ethics approval and consent to participate

The study was performed in accordance with the Declaration of Helsinki. The study was approved by the Regional Committee for Medical and Health Research Ethics (REC) in Norway (REC no. 2012/1679).

## Results

Table 2 summarizes the baseline characteristics of the 433 included patients. Of the 358 compliant patients, 185 (52%) were diagnosed by systematic biopsy, 38 (11%) by mpMRI-targeted biopsy, 75 (21%) by combination (mpMRI-targeted and systematic) biopsy and 60 (17%) by transurethral resection of the prostate (TURP). At diagnosis, 177 (49%) were classified as LR and 181 (51%) as IR. Of the 181 IR patients, 20 (11%) had both GGG 2 tumors and PSA 10–20 ng/ml. IR patients were older and had higher PSA and PSA density compared with LR patients.

**Table 2:** Baseline clinicopathologic characteristics of 417 patients undergoing active surveillance Vestfold Hospital Trust stratified at stratified by compliance with active surveillance protocol and risk group.

Characteristic	Compliant			Non-compliant
	Low-risk	Intermediate-risk	P value^a^	
Patients—no	177	181		59
Median age (IQR)—years	63 (58–68)	66 (61–70)	< 0.001	66 (61–70)
**PSA—no. (%)**			< 0.001	
≤ 6 ng/ml	105 (59)	48 (27)		29 (49)
> 6 ng/ml and ≤ 10 ng/ml	72 (41)	77 (43)		22 (37)
> 10 ng/ml and ≤ 20 ng/ml	0	56 (31)		7 (12)
> 20 ng/ml and ≤ 30 ng/ml	0	0		1 (2)
**Median prostate volume (IQR)—ml**	41 (31–53)	43 (32–57)	0.20	40 (31–55)
Missing—no. (%)	8 (5)	6 (3)		8 (14)
**PSA density (IQR)—ng/ml/cm**^**3**^	0.13 (0.09–0.19)	0.18 (0.13–0.26)	< 0.001	0.15 (0.09–0.22)
Missing—no. (%)	8 (5)	6 (3)		8 (14)
**Gleason grade group—no. (%)**			< 0.001	
1	177 (100)	54 (30)		21 (36)
2	0	127 (70)		18 (31)
3–4	0	0		20 (34)
**Clinical T stage—no. (%)**			0.56	
cT0/pT1	129 (73)	126 (70)		38 (64)
cT2	48 (27)	55 (30)		15 (25)
cT3	0	0		6 (10)
**CAPRA—no. (%)**			< 0.001	
0–2	175 (99)	39 (22)		17 (29)
3–5	0	142 (78)		37 (63)
6–7	0	0		5 (8)
Missing	2 (1)	0		0
**PI-RADS score**^**a,b**^**—no. (%)**			0.25	
≤ 3	27 (15)	22 (12)		3 (5)
> 3	63 (36)	76 (42)		22 (37)
Missing	87 (49)	83 (46)		34 (58)
**Diagnostic procedure type**			0.47	
Systematic biopsy	86 (49)	99 (55)		29 (49)
Targeted biopsy	22 (12)	16 (9)		3 (5)
Systematic and targeted biopsy	36 (20)	39 (22)		15 (25)
TURP^c^	33 (19)	27 (15)		12 (20)
**Median no. of biopsy cores (IQR)**	10.0 (10.0–11.0)	10.0 (10.0–11.0)	0.58	10.0 (10.0–11.0)
Missing—no. (%)	2 (1)	0		0
**Median no. of positive biopsy cores (IQR)**	1.0 (1.0–2.0)	3.0 (1.0–4.0)	< 0.001	3.0 (2.0–4.0)
Missing—no. (%)	2 (0)	0		0
**Median % of positive biopsy cores (IQR)**	16.7 (10.0–25.0)	26.1 (12.5–40.0)	< 0.001	27.3 (16.7–41.7)
Missing—no. (%)	2 (1)	0		0
Maximum tumor extent (IQR)—mm	3.0 (1.0–5.0)	4.0 (2.5–7.0)	< 0.001	5.0 (3.0–9.0)

*CAPRA* cancer of the prostate risk assessment, *IQR* interquartile range, *PI-RADS* prostate imaging–reporting and data system, *PSA* prostate-specific antigen, *TURP* transurethral resection of the prostate.

^a^Fisher's exact (categorical variables) or Mann–Whitney's *U* (continuous variables) test were used to evaluate associations between baseline characteristic and risk group for the compliant patients.

^b^Based on multiparametric magnetic resonance imaging scan performed before diagnostic biopsy.

^c^The tumor percentage was < 5% in 47 (65%) patients and ≥ 5% in 25 (35%) patients diagnosed with TURP.

Note: Percentages may not sum to 100 due to rounding.

**Figure 2:**
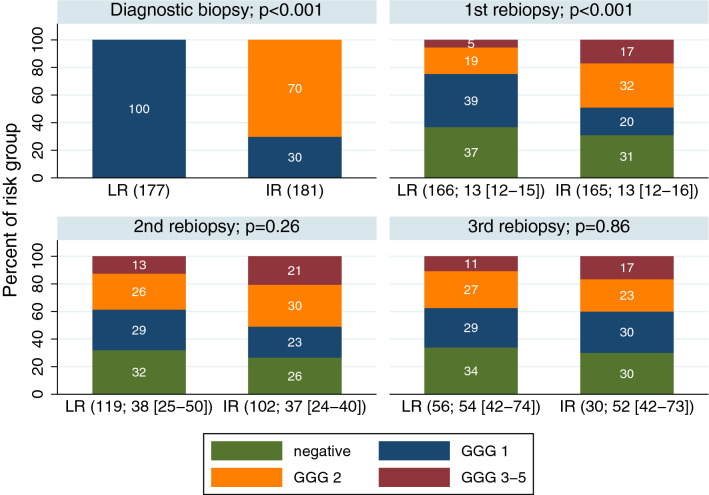
Changes in Gleason grade group with repeat biopsy over time. Number in parentheses represents number of patients and months from diagnosis to a biopsy procedure reported as median with interquartile ranges, respectively. *GGG* Gleason grade group, *LR* low-risk, *IR* intermediate-risk.

Median follow-up was 4.2 years (IQR 2.3–6.0) for all patients and 5.2 years (IQR 3.9–6.7) for patients that did not discontinue AS. During AS, 331 (93%) patients received at least one rebiopsy; 221 had two, 86 had three, 18 had four and seven had five. Of the 663 rebiopsies, 366 (55%) were systematic, 184 (28%) were mpMRI-targeted and 113 (17%) were combination biopsies. Figure 2 depicts changes in GGG for LR and IR patients up till the third rebiopsy. On the first rebiopsy, 29 (18%) of the 165 IR patients were downgraded (13 had GGG 1 and 16 had negative biopsy) and 41 (25%) of the 166 LR patients were upgraded (32 had GGG 2 and 9 to GGG 3–5). The percentage of negative biopsies from the first to the third rebiopsy did not vary much within patient groups, but was marginally higher for LR compared with IR (34–37% vs 26–31%). The percentage of GGG 2 tumors on the first rebiopsy in LR patients was lower compared with IR patients (19% vs 32%). However, this percentage increased considerably and was similar on the second and the third rebiopsy in the two patient groups. The percentage of GGG 3–5 tumors in LR patients was 5% on the first rebiopsy, but doubled on the second and the third biopsy. For IR patients the percentage of GGG 3–5 tumors was more stable over time (17–21%). There was a statistically significant difference in histology outcomes between the two patient groups on the diagnostic- (p < 0.001) and first rebiopsy (p < 0.001), but not on the second- and third rebiopsy (p = 0.26 and p = 0.86, respectively). Similar results were obtained when we studied changes in PSA levels for LR and IR patients (Supplementary Fig. S1); there was a statistically significant difference in PSA levels between the two patient groups on the diagnostic (p < 0.001) and first rebiopsy (p < 0.001), but not on the second- and third rebiopsy (p = 0.57 and p = 0.07, respectively).

Most patients (n = 333, 93%) had a mpMRI scan before or within 1 year after the diagnosis. During AS, 335 patients had at least one mpMRI scan; 255 had two, 109 had three, 33 had four, 17 had five or more. Supplementary Fig. S2 depicts changes in PI-RADS scores for LR and IR patients from the prediagnostic mpMRI scan up till the third surveillance mpMRI scan. We observed similar percentage of PI-RADS ≤ 3 and PI-RADS > 3 tumors in the two patient groups up till the second mpMRI scan (p = 0.25, p = 49 and p = 0.51, respectively). On the third surveillance mpMRI scan, LR patients had significantly higher percentage of PI-RADS > 3 tumor compared with IR patients (89% vs 74%; p = 0.04).

Of the 242 (68%) patients that discontinued AS, 162 (66%) were treated, 64 (26%) were transferred to WW (mostly due to age) and the remaining 16 (7%) were discontinued due to other reasons (Fig. 1). Of the 64 patients transferred to watchful waiting, 21 (33%) were diagnosed by TURP. Treatment was given due to histological upgrading in 107 patients (66%), and/or radiological reclassification in 99 (61%), and/or PSA reclassification in 21 (13%) and/or clinical reclassification in 9 (6%). Only one trigger for treatment was observed in 94 (58%) patients, two in 62 (38%) and three in 6 (4%). Of the 94 patients with one trigger for treatment, 52 (55%) had histological upgrading, 39 (42%) had radiological reclassification and 3 (3%) had PSA reclassification or clinical reclassification.

**Figure 3:**
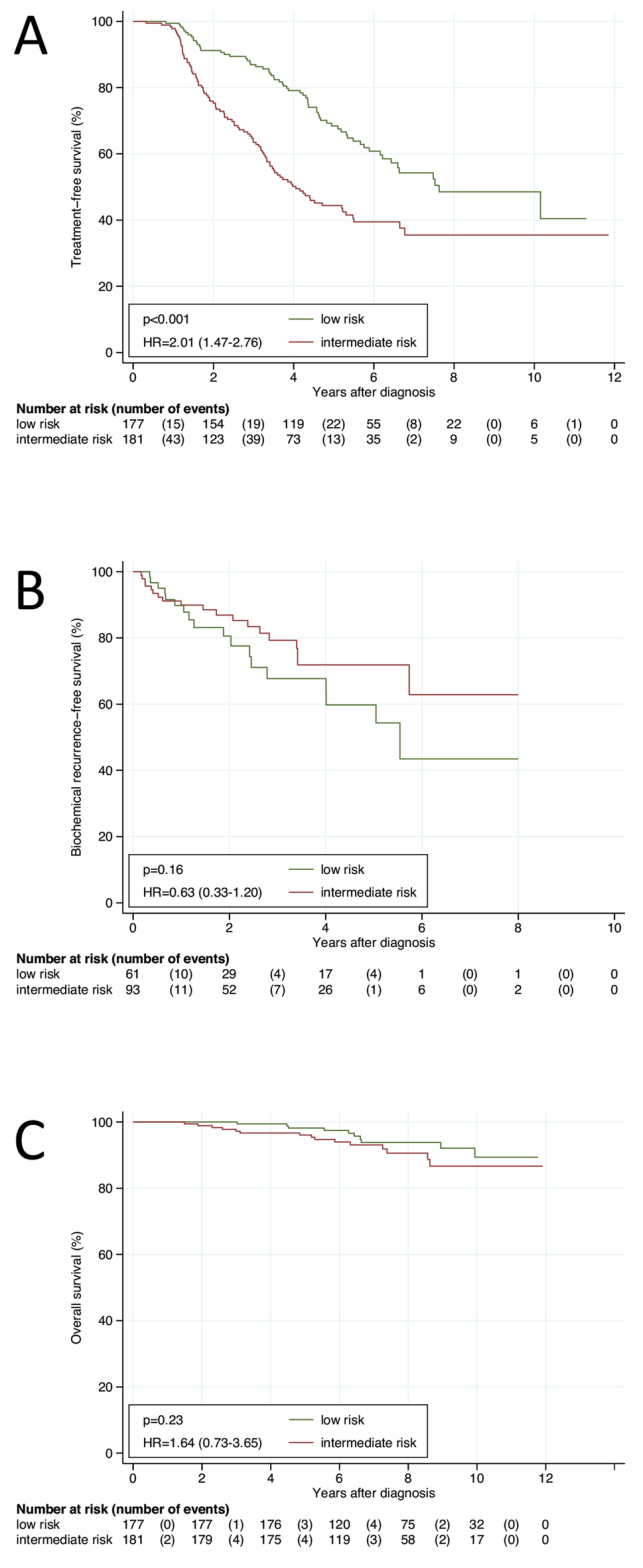
Kaplan–Meier plots of (**A**) treatment-free survival, (**B**) biochemical recurrence-free survival and (**C**) overall survival stratified by low-risk and intermediate-risk group at diagnosis.

Of the 162 treated patients, 65 (40%) were initially LR and 97 (60%) were IR (Fig. 1). Median time to treatment was 2.9 years (IQR 1.6–4.3) for all patients, 3.8 years (IQR 2.3–5.1) for LR patients and 2.3 years (IQR 1.4–3.4) for IR patients. The estimated TFS at 5 years was 56% (95% CI 51–62%). IR patients had higher risk of receiving treatment than LR patients in univariable- (HR 2.01, 95% CI 1.47–2.76, p < 0.001; Fig. 3A) and multivariable analysis (HR 2.21, 95% CI 1.55–3.15, p < 0.001; Table S3). The estimated 5-years TFS was 69% (95% CI 61–76%) for LR patients and 44% (95% CI 36–52%) for LR patients (Fig. 3A).

Most patients were treated with RP (n = 131, 81%; Fig. 1). Nerve-sparing surgery was performed in 69 (59%) of the 118 patients with available data (bilateral in 21 patients and unilateral in 48). Of the 69 patients who received nerve-sparing surgery, 32 were LR and 37 were IR (p = 0.35). The extent of lymph node dissection was similar in both patient groups (median 11 [IQR 8–14] dissected lymphnodes in LR and median 12 [IQR 7–16] dissected lymphnodes in IR, p = 0.28). Table S2 summarizes RP pathology findings for LR and IR patients. AP1 was observed in 89 (71%) of 127 patients and AP2 in 25 (20%) of 126 patients with available data, with no statistically significant differences between patient groups (p = 0.082 and p = 0.82, respectively; Table S2). There were no statistically significant associations between the presence of AP1 and the number of biopsy procedures or mpMRI scans (p = 0.10 and p = 0.72, respectively), and the presence of AP2 and the number of biopsy procedures or mpMRI scans (p = 0.48 and p = 0.21, respectively).

Of the 154 patients with available post-treatment PSA measurements, 37 (24%) developed BCR after a median of 1.5 years (IQR 0.5–2.8). The estimated BCR-free survival at 5 years from treatment was 67% (95% CI 56–76%). In the analysis of BCR-free survival, the HR for IR vs LR patients was 0.63 (95% CI 0.33–1.20, p = 0.16; Fig. 3B) in univariable analysis and 0.51 (95% CI 0.25–1.04, p = 0.06; Table S4) in multivariable analysis. Seven patients were treated with salvage RT.

Two patients developed metastatic disease and three died of PCa. The first patient who died was initially LR and received RP after 5 years of AS due upgrading on rebiopsy to GGG 3 tumor (60% of pattern 4). He experienced BCR only 4 months after RP and progressed on subsequent treatments with antiandrogens, RT and radium-223. He died 9 years after initial diagnosis. The second PCa death occurred in a patient diagnosed with IR disease. After 4 years of AS, mpMRI showed metastatic disease, which was reclassified as sarcoidosis following a biopsy. After further 3 years the patient developed metastases and was treated with androgen deprivation therapy. He rapidly progressed to castrate-resistant disease (< 6 months), received palliative therapy and died within 1 month. The third PCa death occurred in a patient who was initially LR. He was recommended RP after 4 years of AS due to radiological reclassification, but due to an injury was not treated until after 6 mo. Approximately 2.5 years after RP, he developed BCR and died due to treatment-related complications, but no proven metastases, 10 years after the diagnosis.

In total, 25 (7%) patients died of any cause after a median of 5.6 years (IQR 3.1–6.3). The estimated OS at 5 years was 97% (95% CI 95–98%). In the analysis of OS, the HR for IR vs LR patients was 1.64 (95% CI 0.73–3.65, p = 0.23; Fig. 3C) in univariable analysis and 1.25 (95% 0.47–3.31, p = 0.65; Table S5) in multivariable analysis.

Outcomes of the 59 (14%) non-compliant patients who were managed with AS based on their preferences are presented in Supplementary Figs. S1 and S4. In terms of TFS and OS, the non-compliant patients appeared to have intermediate outcomes compared with LR and IR patients (Supplementary Fig. S4).

## Discussion

This study reports a non-academic, population-based AS cohort comprising nearly equal number of LR and selected IR patients. The patients were monitored with regular mpMRI scans in addition to standard measurements, according to the predefined protocol. We observed that IR patients followed clinical and histological trajectories similar to LR patients, with no significant differences in outcome after intermediate term follow-up.

Our study is the first to show changes in GGGs on serial biopsies for both LR and IR patients. In contrast to previous studies, we observed a higher rate of upgrading to GGG ≥ 2 disease for LR patients on the first rebiopsy (13% vs 24%)^24,25^. This is likely due to the more frequent use of mpMRI and targeted biopsies in our cohort, which is known to increase the detection of high-grade tumors^13^. GGG distribution was increasingly similar for LR and IR patients after each round of rebiopsies. Although TFS for IR patients was approximately half compared with LR patients, other outcomes in terms of AP, BCR-free survival and OS did not differ between the two patient groups. This, in addition to the older age and higher PSA values of IR patients at diagnosis, suggests that LR and IR patients in our cohort are diagnosed at different time points on an otherwise similar clinical, histological and prognostic trajectory.

In the recent years, mpMRI and mpMRI-targeted biopsies have been increasingly incorporated into AS protocols; however, their optimal used for patient selection and follow-up are yet to be defined^24,26,27^. In academic AS cohorts including LR and IR patients and median follow-up 4–6.4 years, mpMRI was either irregularly used^15,19,28^ or it replaced repeat biopsies in AS protocol, which were performed in cases of mpMRI, clinical, or PSA progression^28^. We observed higher rates of treatment compared with these studies (45% vs 27–39%)^15,18,19,28^. However, our predefined AS protocol included regular mpMRI scans and mpMRI-targeted biopsies in addition to standard measurements as well as multiple triggers for treatment, and 24% of the patients were treaded solely due to radiological progression. Also, as we included a higher number of patients with GGG 2 tumors compared with previous studies (36% vs 7–22%)^15,18,19,28^, the risk of progression in our cohort may have been higher. Interestingly, we observed statistically significant differences in PI-RADS scores between the two patient groups on the third surveillance mpMRI scan, which could be due to IR patients being treated earlier.

Shorter TFS for IR patients was also reported by Godtman et al.^22^, while no difference in TFS between the two risk groups was observed by others^16,17^. However, in contrast to our cohort, these studies did not use the same triggers for treatment for all patients and patients were not followed by regular mpMRI scans. Interestingly, we observed that LR patients appeared to experience BCR after curative treatment more rapidly than IR patients, but the difference was not statistically significant. The observed trend could be explained by the earlier treatment of IR patients as the histology findings at RP, the number of patients receiving nerve-sparing surgery and the extend of lymphnode dissection was similar in both patient groups. The rate of adverse pathology, when using the AP1 definition, was nearly double than previously reported (71% vs 34%)^17^. This was to be expected given that our treatment triggers were more lenient. Applying the more severe AP2 definition, we observed adverse pathology in 20% of the patients. This definition better corresponded with PCa-specific mortality than the AP1 definition in a study by Kovac et al.^29^. Still, adverse pathology is an intermediate outcome, and the 10-year PCa-specific survival of patients with AP2 in that study was 97% (95% CI 93–100)^29^.

Transferal from AS to WW commonly occurs when patients’ life expectancy is too short to benefit from curative treatment, but its rates are underreported in AS studies^30^. Based on data from the National Prostate Cancer Register of Sweden, Van Hemelrijck et al.^30^ estimated that 48% of patients with LR PCa starting AS were eventually transferred to WW over a life course. This proportion increased with age at time of AS inclusion, with 50% of patients aged 70 years being transferred to WW after 10 years^30^. Published studies with follow-up of 1.8 years to 5.7 years reported the rate of transferal to WW of 3–7%^18,24,28,31,32^, which is substantially lower compared with our cohort (26%). Importantly, 33% of the patients transferred to WW were those diagnosed by TURP, which are generally underrepresented in several AS cohorts^16,19,28^. Patients diagnosed by TURP may be more likely to be transferred to WW than those diagnosed by biopsy; A recent study comparing outcomes in these two patient groups reported that patients diagnosed by TURP experienced disease reclassification significantly later during AS (25% of the patients had disease reclassification within 11.2 years in the TURP group and 3.6 years in the biopsy group), which could be due to partial or complete removal of the transitional zone tumors during TURP^31^. Furthermore, our cohort was followed with a protocol with clearly defined criteria for the transferal to WW. As the age at which patients are transferred to WW was changed from 75 to 80 years during the course of our study, we expect that the rate of transferal to WW will be lower in the future.

 Longer follow-up is needed to draw definitive conclusions regarding metastatic and mortality risks in LR and IR patients^8,9^. In our cohort only two patients developed metastatic disease so far, one in the LR and the other in the IR group. Studies with median follow-up up to 6.4 years reported that patients with IR disease had higher likelihood of developing metastatic disease than those with LR^15,33^. In the Sunnybrook cohort, the estimated metastasis-free survival at 10 years and 15 years was worse in the IR group (defined as PSA 10–20 ng/ml, GGG 2–3 or cT2c) compared with the LR group (10-year 90.7% vs 95.8% and 15-year 82.2% vs 94.6%)^33^. Similarly, the estimated 7-year metastasis-free survival in the University of California cohort was 96% for patients initially diagnosed with GGG 2 and 99% for those with GGG 1 tumors^15^. However, a minority of patients in these studies had received at least one mpMRI scan which likely reduced the detection rates of more aggressive disease.

The inclusion criteria of our AS protocol are one of the widest among the published AS studies^4^. Unlike previous studies, we did not restrict inclusion of IR patients to older or comorbid patients, but used the same age and life expectancy criteria for both LR and IR patients. We reasoned that mpMRI would enable detection of IR disease unsuitable for AS and the remaining IR patients could be followed by AS with a sufficient degree of safety. We used CAPRA score to define risk groups in our study, as it better predicts PCa-specific mortality than the D’Amico and D’Amico-derived systems^34^ and allows risk group substratification. However, we classified patients with GGG 2 or PSA 10–20 ng/ml as IR regardless of the CAPRA score to even better separate the two patient groups. A similar IR definition (CAPRA 3–5 or GGG 2) was previously used in the University of California AS cohort^16^. More recent data implies that selection of IR patients eligible for AS could be further refined by excluding patients with cT2c tumors^35^ and those with intraductal carcinoma or cribriform pattern^36^. These aspects have not been addressed in this study.

The strength of this study is the population-based design reflecting clinical practice and patient preferences. The prospective protocol for follow-up and triggers for treatment allowed for a reasonable comparison between LR and IR when excluding the non-compliant patients. The main limitation of our study is the short follow-up.

## Conclusion

Our study suggests that initial AS combined with mpMRI may be a safe option for selected patients with IR PCa. Despite a high rate of conversion to curative treatment, mpMRI-supported AS appears to be a promising method for selecting IR patients who follow a clinical trajectory comparable to LR patients.

## Supplementary Information

Supplementary Information.

## Data Availability

Norwegian legislation does not allow for data sharing.
