# Supplementary material for: No significant difference in intermediate key outcomes in men with low- and intermediate-risk prostate cancer managed by active surveillance
Source: Sci Rep. 2022 Apr 25;12:6743. doi: 10.1038/s41598-022-10741-8 (PMC9039068; doi:10.1038/s41598-022-10741-8)
Supplement: Supplementary file 1 — Supplementary Information. [file 41598_2022_10741_MOESM1_ESM.pdf]

No significant difference in intermediate key outcomes in men with low- and intermediate-risk prostate cancer managed by active surveillance

Karolina Cyll [1,2], Sven Löffeler [1], Birgitte Carlsen [3], Karin Skogstad [1], May Lisbeth Plathan [1], Martin Landquist [4] and Erik Skaaheim Haug [1,2,*]

[1] Department of Urology, Vestfold Hospital Trust, Tønsberg, Norway, [2] Institute for Cancer Genetics and Informatics, Oslo University Hospital, Oslo, Norway, [3] Department of Pathology, Vestfold Hospital Trust, Tønsberg, Norway, [4] Department of Radiology, Vestfold Hospital Trust, Tønsberg, Norway

*Corresponding author. Department of Urology, Vestfold Hospital Trust, NO–3103 Tønsberg, Norway; Tel.: +47 33 34 33 07; e-mail address: erik.haug@siv.no (E. S. Haug)

Table S1. Patients diagnosed with low- or intermediate-risk disease between 2003 and 2016 at Vestfold Hospital Trust that were enrolled in active surveillance or received immediate treatment (radical prostatectomy or radiotherapy).

Year of diagnosis	Low-risk (GGG 1 and PSA <10ng/ml and cT<T3)		Intermediate-risk (GGG 2 or PSA 10-20ng/ml and cT<T3)	
	Active surveillance	Immediate treatment	Active surveillance	Immediate treatment
**2009**	18	9	8	23
**2010**	19	9	11	20
**2011**	18	3	27	29
**2012**	27	1	22	9
**2013**	21	3	23	19
**2014**	24	0	39	8
**2015**	23	2	31	16
**2016**	32	0	22	15
**Total**	182	27	183	139

Table S2. Associations between risk group at cancer diagnosis and pathology findings at radical prostatectomy

Characteristic	Low-risk no. (%)	Intermediate-risk no. (%)	p-value
**Adverse pathology 1[a], n = 127**			0.082
Absent	21 (38)	17 (24)	
Present	34 (62)	55 (76)	
**Adverse pathology 2[b], n = 126**			0.82
Absent	45 (82)	56 (79)	
Present	10 (18)	15 (21)	
**Gleason grade group, n = 126**			0.78
1	3 (6)	1 (1)	
2	28 (51)	35 (49)	
3	18 (33)	25 (35)	
4	4 (7)	6 (9)	
5	2 (4)	4 (6)	
**Extraprostatic extension, n = 128**			0.28
Absent	34 (62)	37 (51)	
Present	21 (38)	36 (49)	
**Seminal vesicle invasion, n = 128**			0.47
Absent	53 (96)	67 (92)	
Present	2 (4)	6 (8)	
**Lymph node involvement, n = 128**			0.46
Absent	51 (93)	70 (96)	
Present	4 (7)	2 (4)	
**Positive surgical margins, n = 128**			0.70
Absent	37 (67)	52 (71)	
Present	18 (33)	21 (29)	

[a] defined as Gleason grade group ≥3 or pT stage ≥pT3a or pN1; [b] defined as Gleason grade group ≥4 or pT stage ≥ pT3b or pN1

Table S3. Uni- and multivariable cause-specific analyses of treatment-free survival with patient characteristics at prostate cancer diagnosis

Variable	Group	Univariable analysis		Multivariable analysis*	
		HR (95% CI)	p-value	HR (95% CI)	p-value
**Risk group at diagnosis**	Intermediate-risk	2.01 (1.47-2.76)	**<0.001**	2.21 (1.55-3.15)	**<0.001**
**Age at diagnosis**	10-year increment	1.11 (0.86-1.44)	0.43	1.08 (0.83-1.43)	0.56
**PSA**			**0.017**	omitted	
	≤6 ng/ml	Ref.			
	>6 ng/ml and ≤10 ng/ml	1.45 (1.03-2.04)			
	>10 ng/ml and ≤20 ng/ml	1.81 (1.6-2.83)			
**Gleason grade group**	2 vs 1	2.02 (1.48-2.76)	**<0.001**	omitted	
**Prostate volume**	10-ml increment	0.83 (0.76-0.91)	**<0.001**	0.83 (0.75-0.91)	**<0.001**
**Clinical T stage**	cT2 vs cT0/1	1.29 (0.92-1.82)	0.14	0.99 (0.69-1.41)	0.96
**Fraction of positive biopsy cores**	10-% increment	1.09 (1.00-1.18)	**0.048**	0.96 (0.86-1.06)	0.43
**Maximum tumor extent**	2-mm increment	1.20 (1.09-1.32)	**<0.001**	1.15 (1.03-1.29)	**0.011**

Abbreviations: CI = confidence interval; HR = hazard ratio; PSA = prostate-specific antigen.

*Of the 358 patients, 291 (150 treated and 141 not treated) had complete data and were included in the multivariable analysis.

Table S4. Uni- and multivariable cause-specific analyses of biochemical recurrence with patient characteristics at radical prostatectomy

Variable	Group	Univariable analysis		Multivariable analysis*	
		HR (95% CI)	p-value	HR (95% CI)	p-value
**Risk group at diagnosis**	Intermediate-risk	0.63 (0.33–1.20)	0.16	0.51 (0.25-1.04)	0.06
**Age at surgery**	10-year increment	1.26 (0.69-2.27)	0.45	0.85 (0.41-1.76)	0.66
**PSA**			**0.037**		0.43
	≤6 ng/ml	Ref.		Ref.	
	>6 ng/ml and ≤10 ng/ml	1.75(0.59-5.15)		1.20 (0.39-3.64)	
	>10 ng/ml and ≤20 ng/ml	2.45 (0.90-6.65)		2.05 (0.72-5.85)	
	>20 ng/ml	5.85 (1.56-21.92)		2.07 (0.45-9.61)	
**Gleason grade group**			**0.023**		0.29
	2	Ref.		Ref.	
	3	2.64 (1.25 -5.55)		2.09 (0.93-4.71)	
	4-5	2.02 (0.69-5.86)		1.10 (0.35-3.49)	
**Extracapsular extension**	Present vs. Absent	1.37 (0.71-2.65)	**0.35**	1.02 (0.45-2.31)	0.96
**Surgical margins**	Positive vs. Negative	2.41 (1.25-4.64)	**0.007**	1.91 (0.93-3.94)	0.08
**Seminal vesicle invasion**	Present vs. Absent	4.05 (1.66-9.87)	**<0.001**	2.36 (0.74-7.57)	0.15
**Lymph node involvement**	Present vs. Absent	3.83 (1.58-9.29)	**0.001**	2.21 (0.75-6.55)	0.15

Abbreviations: CI = confidence interval; HR = hazard ratio; PSA = prostate-specific antigen.

*Of the 131 patients treated with radical prostatectomy, 122 (35 had biochemical recurrence and 87 did not have biochemical recurrence) had complete data and were included in the multivariable analysis.

Table S5. Uni- and multivariable cause-specific analyses of overall survival with patient characteristics at prostate cancer diagnosis

Variable	Group	Univariable analysis		Multivariable analysis*	
		HR (95% CI)	p-value	HR (95% CI)	p-value
**Risk group at diagnosis**	Intermediate-risk	1.64 (0.73–3.65)	0.23	1.25 (0.47-3.31)	0.65
**Age at diagnosis**	10-year increment	2.87 (1.32-6.23)	**0.008**	2.71 (1.18-6.27)	**0.019**
**PSA**			**0.04**	omitted	
	≤6 ng/ml	Ref.			
	>6 ng/ml and ≤10 ng/ml	3.19 (1.17-8.68)			
	>10 ng/ml and ≤20 ng/ml	1.50 (0.36-6.29)			
**Gleason grade group**	2 vs 1	2.19 (0.99-4.88)	**0.047**	omitted	
**Prostate volume**	10-ml increment	0.79 (0.61-1.02)	0.07	0.74 (0.54-1.00)	0.054
**Clinical T stage**	cT2 vs cT0/1	1.88 (0.84-4.19)	0.12	1.53 (0.62-3.75)	0.36
**Fraction of positive biopsy cores**	10-% increment	1.08 (0.86-1.35)	0.52	0.94 (0.72-1.23)	0.64
**Maximum tumor extent**	2-mm increment	1.19 (0.92-0.15)	0.19	1.15 (0.86-1.54)	0.34

Abbreviations: CI = confidence interval; HR = hazard ratio; PSA = prostate-specific antigen.
*Of the 358 patients, 291 (20 were dead and 271 were alive) had had complete data and were included in the multivariable analysis.

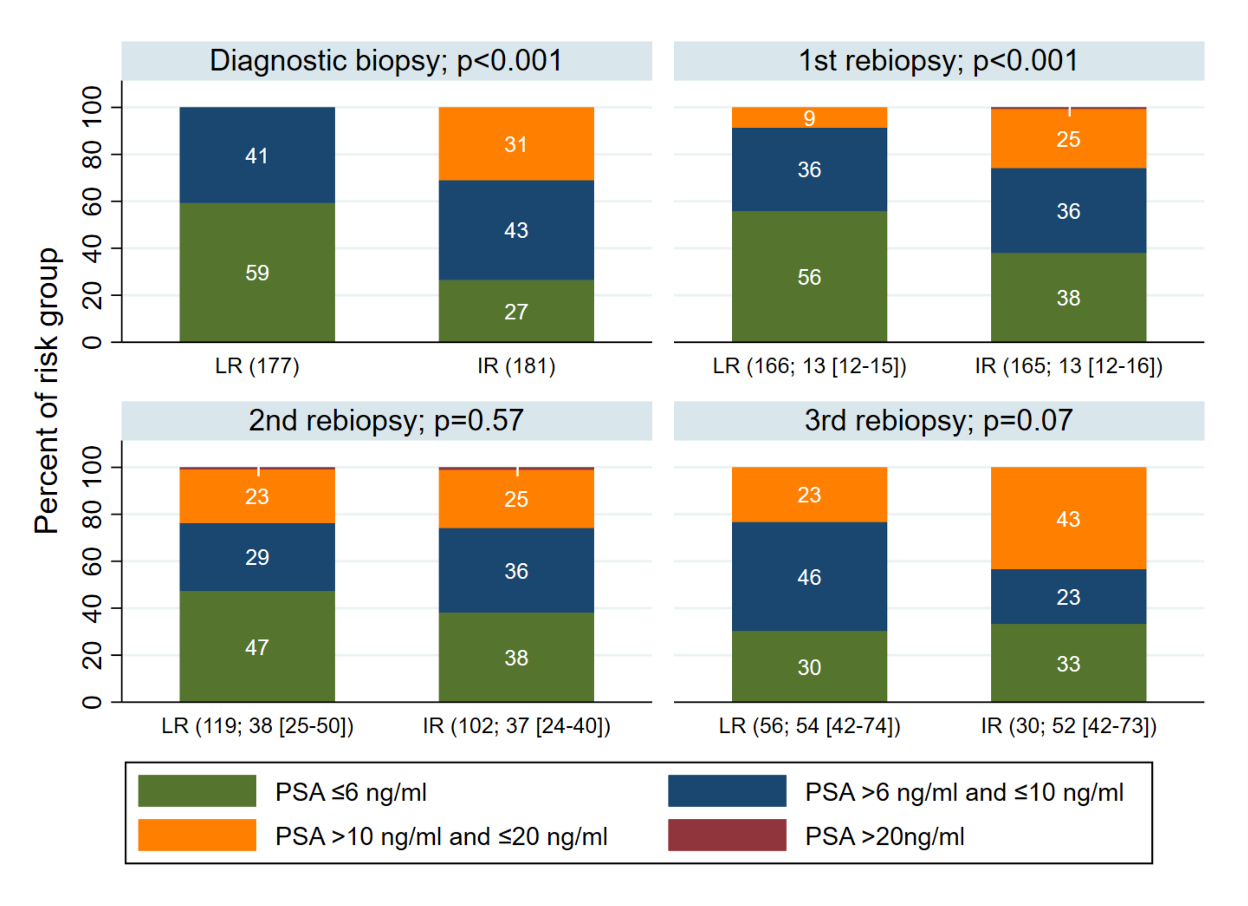

Supplementary Fig. 1 Changes in prostate specific antigen (PSA) levels with repeat biopsy over time. Number in parentheses represents number of patients and months from diagnosis to a biopsy procedure reported as median with interquartile ranges, respectively.
LR = low-risk; IR = intermediate-risk.

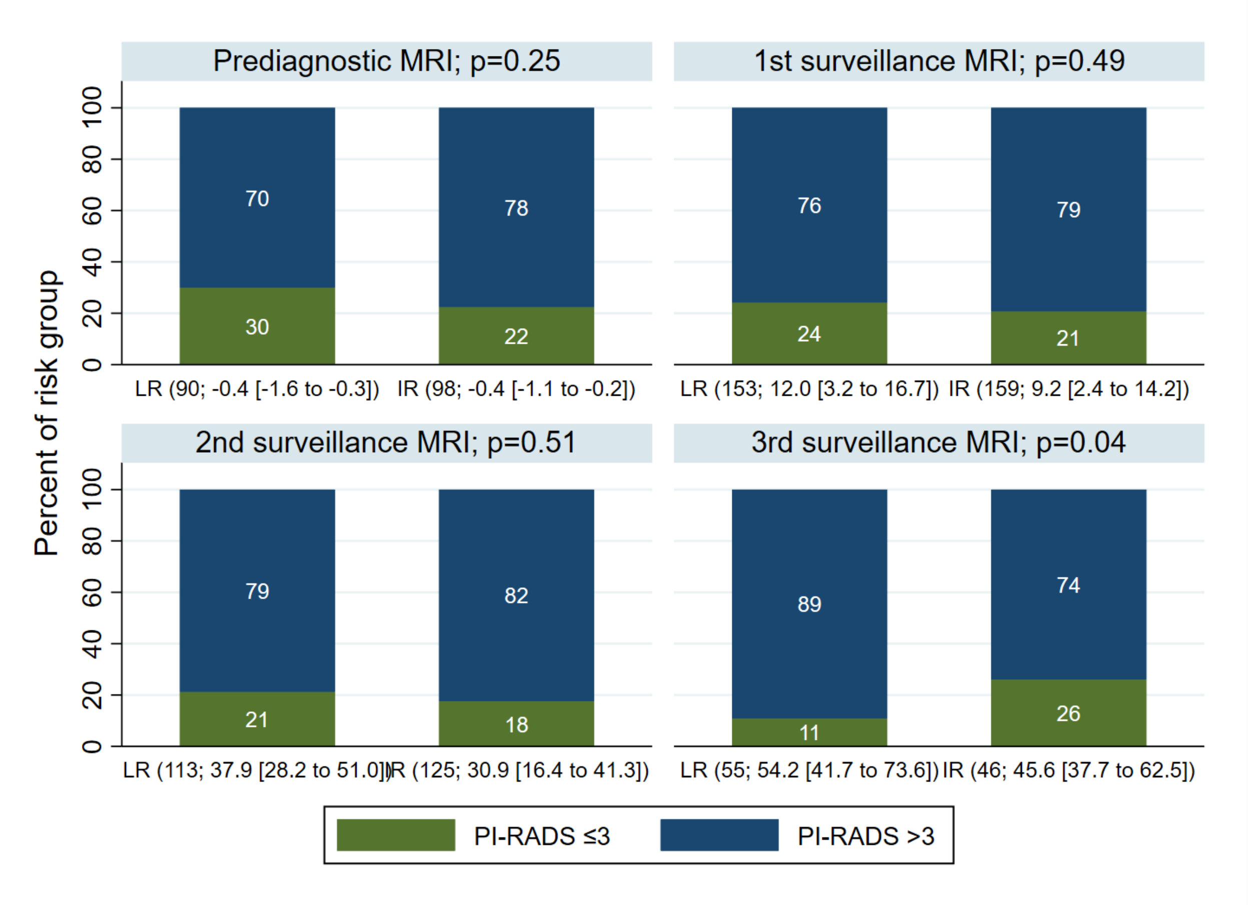

Supplementary Fig. 2 Changes in Prostate Imaging–Reporting and Data System (PI-RADS) scores with repeat multiparametric magnetic resonance imaging (mpMRI) scans over time. Number in parentheses represents number of patients and months from diagnosis to a biopsy procedure reported as median with interquartile ranges, respectively.

NOTE: PI-RADS score was missing for 12 patients at the prediagnostic MRI, 23 at the 1st surveillance MRI, 17 at the 2nd surveillance MRI and 8 at the 3rd surveillance MRI.

LR = low-risk; IR = intermediate-risk.

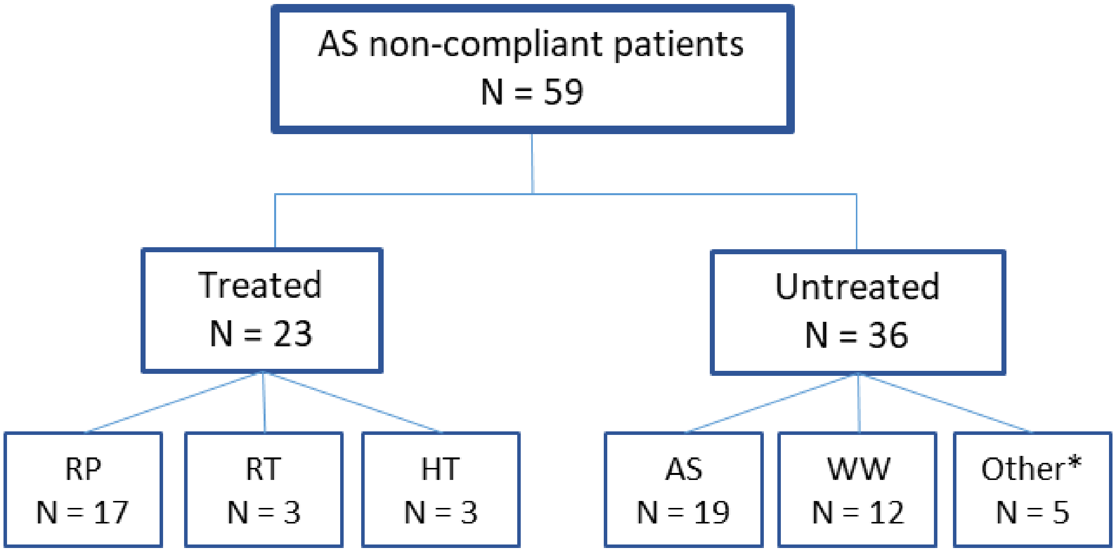

Supplementary Fig. 3 Status of the non-compliant AS patients at the end of follow-up. Median follow-up was 4.3 years (interquartile range [IQR] 1.9–7.6 years). The estimated treatment-free survival at 5 years was 59% (95% confidence interval [CI] 44%–71%). Of the 14 patients with available data following radical prostatectomy, adverse pathology was observed in 10 (71%) patients when it was defined as Gleason grade group (GGG) ≥3 or pT stage ≥pT3a or pN1 and in 6 (43%) patients when it was defined as GGG ≥4 or pT stage ≥pT3b or pN1. Biochemical recurrence was observed in 3 (15%) of the 20 patients with available post-treatment prostate specific antigen (PSA) measurements. Of the five patients who died, one died due to prostate cancer. At diagnosis, he was classified as intermediate risk. After four years, there was an indication of cT3 disease, but the patient chose to continue AS. A rebiopsy performed two years later showed Gleason grade group 3 disease, but the patient could not be treated due to comorbidity. Metastatic disease was confirmed four years after that, and the patient died 12 years after the diagnosis.

*Four patients were lost to follow-up and one died.

Abbreviations: AS = active surveillance; HT = hormonal therapy; RP= radical prostatectomy; RT= radiotherapy; WW = watchful waiting.

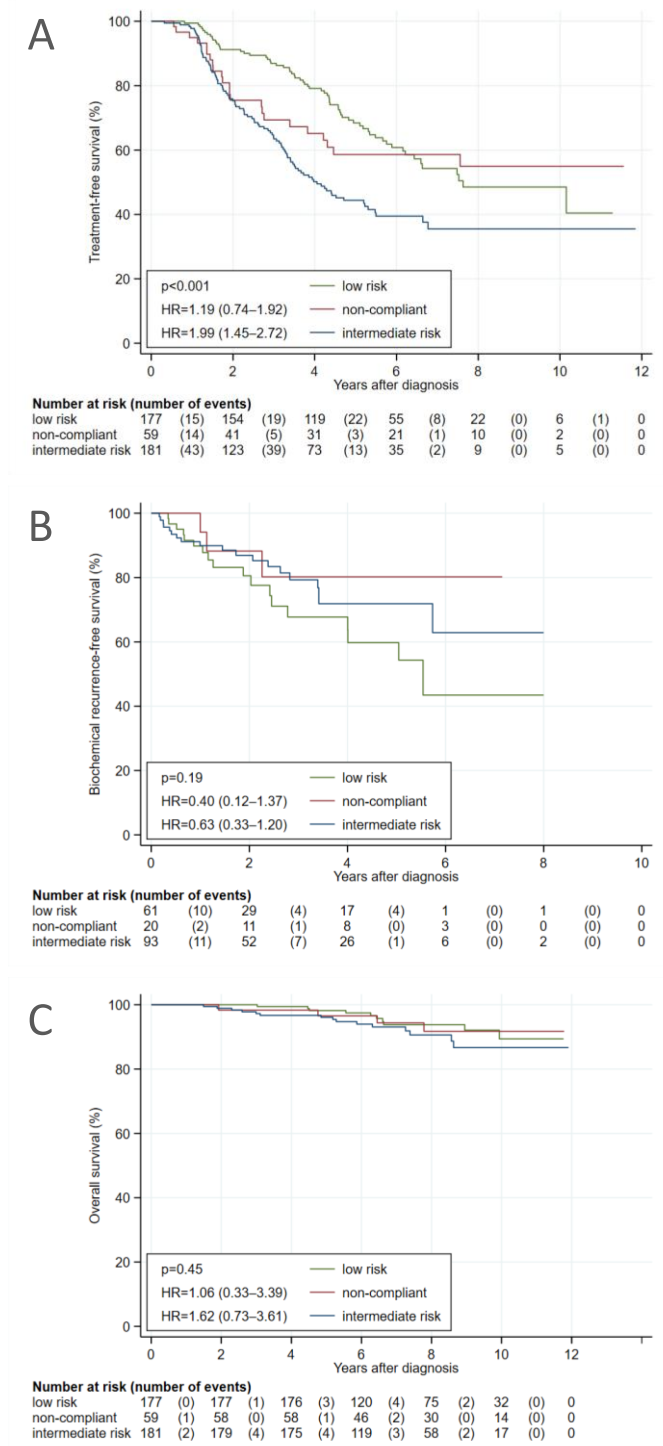

Supplementary Fig. 4 Kaplan-Meier plots of **(A)** treatment-free survival, **(B)** biochemical recurrence-free survival and **(C)** overall survival stratified by low-risk, non-compliant and intermediate-risk group at diagnosis.
